# Bioactivity and Bioavailability of the Major Metabolites of *Crocus sativus* L. Flower

**DOI:** 10.3390/molecules24152827

**Published:** 2019-08-02

**Authors:** Natalia Moratalla-López, María José Bagur, Cándida Lorenzo, M.E. Martínez-Navarro, M. Rosario Salinas, Gonzalo L. Alonso

**Affiliations:** Cátedra de Química Agrícola, ETSI Agrónomos y de Montes, Universidad de Castilla-La Mancha, Campus Universitario, 02071 Albacete, Spain

**Keywords:** crocetin esters, picrocrocin, safranal, kaempferols, anthocyanins

## Abstract

*Crocus sativus* L. has been cultivated throughout history to obtain its flowers, whose dried stigmas give rise to the spice known as saffron. Crocetin esters, picrocrocin, and safranal are the main metabolites of this spice, which possess a great bioactivity, although the mechanisms of action and its bioavailability are still to be solved. The rest of the flower is composed by style, tepals, and stamens that have other compounds, such as kaempferol and delphinidin, which have an important antioxidant capacity, and these can be applied in foods, phytopharmaceuticals, and cosmetics. The aim of this work was to provide an updated and critical review of the research on the main compounds of *Crocus sativus* L. flower, including the adequate analytical methods for their identification and quantification, with a focus on their bioactivity and bioavailability.

## 1. The Plant of *Crocus sativus* L.

**Figure 1:**
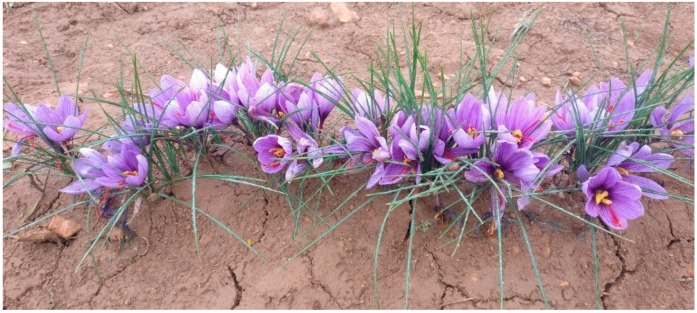
Plant of *Crocus sativus* L.

*Crocus sativus* L. (*C.s.*) is a perennial stemless plant from the family of Iridaceae, vegetatively propagated by corms. This plant remains dormant during summer, and flowering occurs in autumn when all other plants prepare to protect themselves against the rigors of the winter. In the flower of *C.s.*, petals and sepals are undifferentiated and are called tepals. Their flowers have six tepals, three yellow stamens, and a white filiform style culminated in a red stigma divided into three threads (Figure 1).

The genus *Crocus* comprises about 160 species occurring in Europe, the Middle East, and North Africa and is mainly used as ornamentals all over the world for their colorful flowers [1]. *C.s.* is cultivated almost exclusively for its stigma, the spice being the dried stigmas of *C.s.* blossoms. The greatest worldwide producer at the present time is Iran, providing nearly 90% of the world´s total production of saffron [2]. India, Greece, Morocco, Spain, Italy, and Turkey are also involved in the production of saffron. On a smaller scale, small productions are also found in countries like France, Switzerland, China, Afghanistan, Azerbaijan, Japan, Tasmania, New Zealand, Argentina, Mexico, the United States, United Kingdom, and Portugal [1].

For centuries, the cultivation of *C.s.* has required a large amount of labor during a relatively short period of time. This means that this crop was reduced and even abandoned in several countries through its history. However, at present, mechanization and modernization of traditional production methods have been introduced. Currently, there are various machines to collect saffron flowers [3,4]. These machines have been used in the last harvests, obtaining good results in terms of operation and collection of *C.s.* flowers even on wet ground. In addition, several producers are implementing forced production under greenhouses and controlled microclimatic conditions. Thus, the mechanization and modernization of saffron production offers the opportunity to increase again its cultivation, collecting a large number of *C.s.* flowers in shorter time and obtaining them for a longer period of time than the traditional cultivation.

In traditional saffron production, the stigma is separated from the rest of the *C.s.* flower, and the remaining parts (tepals, stamens, and style) constitute an agriculture bioresidue. About 93 g per 100 g of *C.s.* flower are floral bioresidues and about 63 kg of these bioresidues are generated to produce 1 kg of saffron [5]. The tepals are the parts that contribute most to the mass of the *C.s.* flower with 78.4%, followed by stamens with 13.4%, stigmas with 7.4%, and styles with 0.7%. Therefore, once the stigmas are separated from the *C.s.* flowers, a large amount of floral bioresidues is obtained, which accounts for 92.6% of the mass of *C.s.* flowers.

Saffron is one of the most appreciated spices in the world. It is the only spice able to transmit color, flavor, and aroma to foods. Crocetin esters are responsible for the red color of this spice and for its coloring capacity to give yellowish-red hues. Picrocrocin is the foremost contributor to the bitter taste characteristic, and safranal is the major compound in the saffron volatile fraction contributing to its aroma [6].

Apart from crocetin esters, picrocrocin and safranal, saffron possesses other bioactive compounds, such as kaempferols and its glycosides [1]. Kaempferols are gaining increasing interest for their antioxidant activity as a food supplement, in functional foods, in pharmaceutical preparations, and cosmetic formulations [7]. The perianth of *C.s.* flowers (tepals) possesses high-phenolic content that is mainly made of anthocyanins and flavonols [8]. Kaempferol glycosides are the major flavonoids in such flowers. These represent 70%–90% of the total content in the perianth, and they are also present in their leaves [1]. In stigmas, kaempferol glycosides are the main flavonoids, whereas a methylated kaempferol, kaempferide, and isorhamnetin glycosides are detected in pollen [9]. In addition to kaempferol and kaempferol glycosides, there are other flavonols in the leaves of *C.s*., such as quercetin and flavones (luteolin, tricin, acacetin, apigenin, and scutellarein), and their glycosides are also presented [10]. Anthocyanins are responsible for the attractive color of tepals, among which delphinidin, petunidin, and malvidin glycosides represent 30% of the total content of phenolic compounds in the perianth [11].

It is known that flavonoids are commonly found in plants and constitute a significant part of the human diet [12]. A flavonol-rich diet is linked with a relatively low occurrence of degenerative diseases and various forms of cancer [7]. Regarding *C.s.*, their flowers have been traditionally consumed as sweets in Sardinia (Italy) [5]. On the other hand, in the current cuisine, using edible flowers to decorate dishes has become more usual. This, together with the fact that it is currently possible to perform the mechanization and modernization of saffron crop, has led to a growing interest in the development of new food products from *C.s.* flower beyond saffron. Hence, apart from bioactive compounds of stigmas, floral bioresidues of *C.s.*, as valuable natural sources of antioxidants, have been employed as active ingredients in high-end cosmetic products [13], and they could contribute to the development of new food products [14].

Thus, the use of the whole flower of *C.s.* can be contemplated as a new ingredient in the food industry and in phytopharmacy. With this purpose, the aim of this work was to provide an updated and critical review of the research on the main compounds of *Crocus sativus* L. flower, including the adequate analytical methods for their identification and quantification, with a focus on their bioactivity and bioavailability.

## 2. Analytical Methods for *Crocus sativus* L.

**Figure 2:**
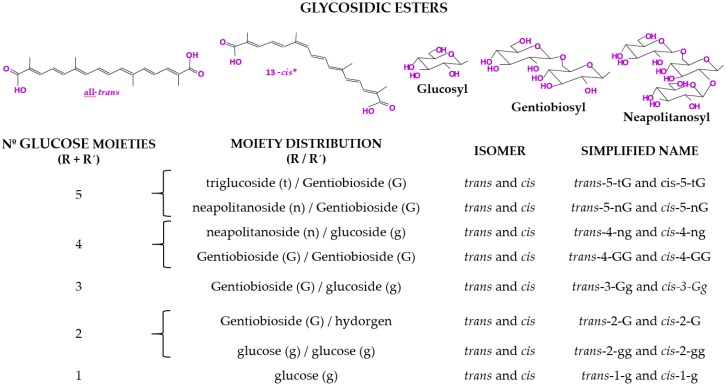
Structure and simplified name of the crocins (glycosidic esters of crocetin) found in saffron [6].

Saffron is highly valued for providing color, taste, and a characteristic aroma to the foods and beverages to which it is added. The quality of saffron in international commercial agreements is determined according to the ISO 3632:2011 standard [15] that classifies saffron into three categories upon their physical and chemical characteristics. Color is the most important quality characteristic [6]. A group of water-soluble carotenoids derived from crocetin (8,8′-diapo-Ψ,Ψ′-carotenedioic acid, C_20_H_24_O_4_) are responsible for the coloring capacity of this spice (Figure 2). Glucose, gentiobiose, and neapolitanose are the sugars that esterify the end of the crocetin, which gives the crocetin esters, the geometric isomers *trans* being the most abundant ones and the *cis* isomers the minority [16]. Crocins are the other way to appoint these carotenoids. The first reference to crocin was made by Aschoff in 1818, who gave it its name [17]. The content of crocetin esters represents 16%–28% of saffron, and some harvest years, it can reach up to 30% [18]. Due to the importance of this group of carotenoids, its kinetics in saffron aqueous extracts upon thermal treatment in darkness have been studied [19].

**Figure 3:**
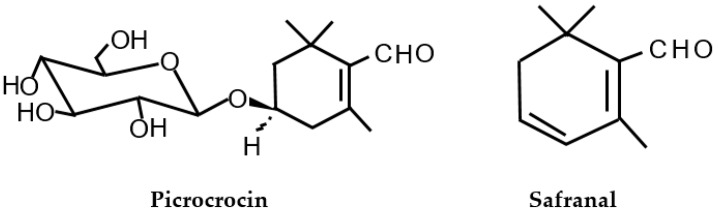
Structures of picrocrocin and safranal found in saffron [6].

Picrocrocin (4-(*β*-*D*-glucopyranosyloxy)-2,6,6-trimethyl-1-cyclohexene-1-carboxaldehyde, C_16_H_26_O_7_) is thought to be the foremost contributor to the bitter taste of saffron (Figure 3) [6,20,21]. This compound has been identified only in the genus *Crocus*, of which the only edible species is *C.s.* Hence, picrocrocin is a molecular marker of this spice, whose taste cannot be imitated from other spices or seasonings, and it can serve to identify true saffron [22]. This compound was isolated for the first time in *C.s.* stigmas in 1922, and its molecular structure was defined in 1934 [23,24]. Several glycosidic compounds, which are related to picrocrocin, have also been identified [6,25,26,27]. The picrocrocin content ranges between 7%–16% in the dried stigmas of *C.s.* and it is possible to find samples that have reached up to 20% [28]. The picrocrocin stability in aqueous extracts of saffron upon thermal treatment was determined by Sánchez et al. [29], this compound being more stable than the crocetin esters.

Related to the volatile fraction of saffron, more than 40 compounds have been identified [25,30,31,32,33], safranal (2,6,6-trimethyl-1,3-cyclohexadiene-1-carboxaldehyde, C_10_H_14_O) being the major compound which contributes to its aroma (Figure 3) [34,35,36,37,38]. Safranal is the aglycone of picrocrocin [6]. Picrocrocin is converted to safranal either by a two-step enzymatic/dehydration process involving the intermediate HTCC (4-hydroxy-2,6,6-trimethyl-1-cyclohexen-1-carboxaldehyde, C_10_H_16_O_2_) or directly by thermal degradation [6,22,39]. This compound has been detected in very few plants, and it can be generated when certain carotenoids are subjected to a thermal process [40,41]. The safranal concentration in saffron is usually 0.1%–0.6% [42]. In the saffron of Protected Designation of the Origin (POD) “Azafrán de La Mancha”, which possesses the highest quality in the world, safranal represents over 65% of the total aroma, expressed as a percentage of total content in volatile substances, determined by gas chromatography (GC–SPME) [43].

There is no other spice which possesses such a high content of crocetin esters and picrocrocin [18]. Today, the saffron quality continues to be assessed according to ISO 3632:2011 [15] standard, in which the three foremost parameters (color, taste, and aroma) are determined by UV-vis spectrophotometry. Coloring strength ($E_{1\;cm}^{1\%}$440 nm) is the most important characteristic in saffron quality; therefore, the values obtained from its aqueous extracts are critical for the commercial value of this spice. The $E_{1\;cm}^{1\%}$ at 440 nm is directly correlated with the ability of transmitting color to foodstuffs to which saffron is added [44]. $E_{1\;cm}^{1\%}$440 nm values of saffron depend on the crocetin esters content in the dried stigma of *C.s.* These values serve to classify saffron into categories, but it is not possible to determine by UV-vis spectrophotometry the amount of crocetin esters that is included in a saffron sample, and neither is it possible to obtain the ratio of *trans*/*cis* crocins or the concentration of them.

The other parameters are $E_{1\;cm}^{1\%}$257 nm and $E_{1\;cm}^{1\%}$330 nm. With these parameters, it is intended to assess picrocrocin and safranal, respectively, but in fact, the values of $E_{1\;cm}^{1\%}$257 nm and $E_{1\;cm}^{1\%}$330 nm only serve to classify saffron into categories since, although in ISO 3632:2011, it is indicated that 257 nm is the wavelength at which picrocrocin has its maximum absorbance, and at 330 nm, safranal possesses its maximum absorbance, there are other compounds that also adsorb at this wavelength. Thus, at 250 nm, in an aqueous extract of saffron, there are other substances such as *trans* crocetin esters that also adsorb, and *cis* crocetin esters also adsorb at 250 and at 330 nm. Therefore, the parameters $E_{1\;cm}^{1\%}$257 nm and $E_{1\;cm}^{1\%}$330 nm do not give an accurate measurement of picrocrocin and safranal, respectively, and spectrophotometric measurements according to ISO 3632:2011 [15] lead to erroneous results or can lead to a misinterpretation of the results obtained. Thus, using ISO 3632:2011 (Part 2) [15] without knowing that it is the wrong standard has meant that today, there exists a global confusion around the chemical characteristics of the saffron spice. Hence, this misunderstanding of Part 2 has been extended to other official country standards, Protected Designations of the Origin (PDOs), and Codex Alimentarius. Moreover, the overestimation of picrocrocin and safranal determined by UV-vis according to ISO 3632:2011 could be the cause of significant errors in the products elaborated with these compounds [18]. For this reason, more accurate methods for determination of the crocetin esters, picrocrocin, and safranal have been developed, and they are being continuously revised.

Currently, there is a wide range of analytical techniques used for saffron analysis (Table 1). Saffron quality should be determined by methods which provide detailed quantification of the crocetin esters, picrocrocin, safranal, and other compounds of interest. Today, there are different techniques with which the identification and quantification of the main compounds of saffron is possible. In addition, normally, these techniques use methods that have been validated using standards of the metabolites of interest, which allows the determination of the compounds to be very accurate [45].

To determine crocetin esters and picrocrocin, liquid chromatography method equipment with an aligned diode detector (HPLC–DAD) may be used from aqueous extracts of saffron [46,47]. To determine the volatile fraction of this spice, different analytical techniques have been developed, such as gas chromatography/mass spectrometry (GC–MS) and olfactometry [48], GC–MS [38,49], HPLC–DAD [24,39], HPLC–MS/MS [50] or ultrasound-assisted extraction by UV-vis [51]. If only safranal needs to be identified and quantified, HPLC–DAD can be used, as proposed in a specific approach carried out by García-Rodríguez et al. [46]. In addition, the correlation between different techniques used for saffron analysis has also been studied, which found that there was no correlation between the safranal content obtained by HPLC–DAD and the ISO 3632:2011 standard when saffron samples from different origin were analyzed [42]. In this sense, no relationship was found between $E_{1\;cm}^{1\%}$330 nm and safranal content analyzed either by HPLC–DAD or by GC, due to the interferences from *cis*-crocins mentioned above [52]. Therefore, HPLC–DAD is adequate for determining the three main parameters that define the saffron quality, and this approach could be included in the ISO 3632:2011 method [42,46].

On the other hand, it is worth noting the nuclear magnetic resonance (NMR) spectroscopy technique due to increasing interest in the last years [53]. This technique has been employed as a useful method for plant metabolite fingerprinting, with the purpose of sample classification and the determination of discriminatory features [54]. NMR spectroscopy offers the possibility to detect several classes of chemical compounds simultaneously [53]. In saffron, the NMR-based metabolomics approach was employed for the first time in 2010 with the aim of obtaining the metabolite fingerprinting of Iranian saffron [55]. Thus, the metabolite fingerprinting of saffron can be used to distinguish authentic saffron from others, and in addition to that, NMR is employed today with a particular focus on geographical origin characterization, aging determination, and fraud detection [54,56,57].

Regarding phenolic compounds, in dried stigmas of *C.s.*, some kaempferols glycosides have been found [58]. Flavonols are a subgroup of a larger group of structurally-related compounds, the flavonoids, whose basic structure consists of two phenyl groups joined by a three-carbon bridge [67]. The glycosides are compounds produced during the secondary metabolism of plants that are characterized by having two distinct parts in their molecule: on the one hand, a sugar, and on the other hand, an organic molecule, both linked by alpha (*α*) or beta (*β*) glycosidic bonds. The organic molecule is called aglycone, it can belong to very diverse chemical families, and it is the reactive part of the glycoside, but its union with sugar allows it to increase its solubility in water, and therefore its translocation and absorption, since the solubility in water of the aglycones is limited [68]. During the secondary metabolism, firstly, the aglycones are formed, and then the glycosylation takes place, which is carried out by the enzymes called glycosyltransferases. These enzymes are located in the cytosol of the plant cells. From the cytosol, the glycosides are translocated to other plant organs, where they accumulate and produce their function [69]. Thus, the aglycones are active or “bioactive” molecules with a defined mission that they must perform in the relevant organs of the plants, and to fulfill it, they have to be glycosylated, since it is the form in which they are soluble in the medium of transport.

**Figure 4:**
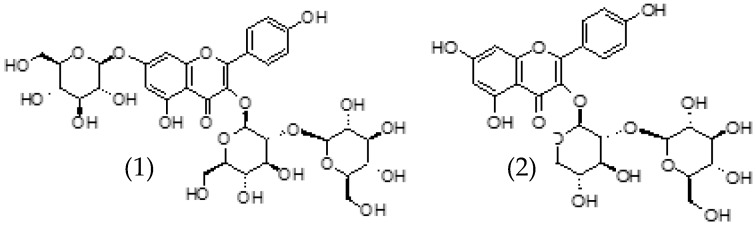
Structures of kaempferol 3-*O*-*β*-sophoroside 7-*O*-*β*-glucoside (1) and kaempferol 3-*O*-*β*-sophoroside (2) found in *Crocus sativus* L. [1].

Kaempferol 3-*O*-*β*-sophoroside-7-*O*-*β*-glucoside is the most important flavonol in saffron (Figure 4). This kaempferol was first isolated from saffron by Straubinger et al. [70]. Its relative content ranges from 37% to 63% of total flavonoids, and its absolute content values vary between 1.47 and 2.58 of the equivalent mg of rutin g^−1^ [58]. In order of importance, related to the concentration, kaempferol 3-*O*-*β*-sophoroside is the next major flavonol in this spice (Figure 4). Its relative content ranges from 16% to 47% of total flavonoids, and its absolute content values vary between 0.61 and 3.12 of the equivalent mg of rutin g^−1^ [58]. Kaempferol 3,7,4′-tri-*O*-*β*-glucoside is another flavonol detected in saffron. Its relative content ranges from 16% to 22% of total flavonoids, and its absolute content values ranges from 0.59 to 1.09 of the equivalent mg of rutin g^−1^ [58]. Kaempferol 7-*O*-*β*-sophoroside was also isolated for the first time from the saffron by Straubinger et al. [70].

As for the floral bioresidues of *C.s*., particular attention is given to its tepals, which are rich in flavonols. Saffron tepals are one of the richest sources of kaempferol and its glycosides. The level of this flavonol in saffron tepals, in g/kg, is about 100 higher than in foods considered “rich” in kaempferol [71]. As mentioned above, this part of such a flower also possesses anthocyanins. Further, stamens and styles also have phenolic compounds. Therefore, apart from stigmas, the tepals, stamens, and styles of flowers are a natural source of antioxidants and active principles [14].

There are seven kaempferols glycosides, plus its kaempferol aglycone, which have been reported in floral bioresidues of saffron. Moreover, quercetin 3-*O*-*β*-sophoroside and isorhamnetin 3,4´-tri-*O*- *β*-glucoside, along with five anthocyanins, have also been reported in these floral bioresidues [11,72,73]. Kaempferols, in decreasing order according to their concentration, are as follows: kaempferol 3-*O*-*β*-sophoroside, kaempferol aglycone, kaempferol 3-*O*-*β*-sophoroside-7-*O*-*β*-glucoside, kaempferol 3-*O*-*β*-rutinoside, kaempferol 3,7-di-*O*-*β*-glucoside, kaempferol 7-*O*-*β*-glucoside, kaempferol 3,7,4′-tri-*O*-*β*-glucoside and kaempferol 3-*O*-*β*-glucoside. With regard to anthocyanins, these are as the following: delphinidin 3,5-di-*O*-*β*-glucoside, petunidin 3,5-di-*O*-*β*-glucoside, delphinidin 3-*O*-*β*-glucoside, malvidin 3,5-di-*O*-*β*-glucoside, and petunidin 3-*O*-*β*-glucoside.

Kaempferol 3-*O*-*β*-sophoroside is the main flavonoid in the *C.s.* flowers. This flavonol was extracted from saffron floral bioresidues that were mainly made up of tepals, and an extract yield of 2.3 mg g^−1^ dry weight was obtained [74]. Various authors have studied the content of this kaempferol in tepals. According to Goupy et al. [72], kaempferol 3-*O*-*β*-sophoroside represents about 55% of total flavonoids, and its content ranges from 0.69 to 12.60 mg equivalent of kaempferol 3-*O*-*β*-glucoside g^1^ dry weight [11,72,73].

**Figure 5:**
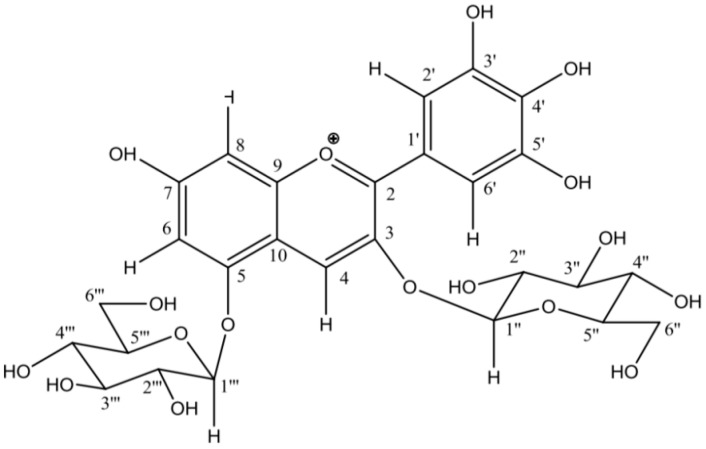
Structure of delphinidin 3,5-di-*O*-*β*-glucoside found in *Crocus sativus* L.

Delphinidin 3,5-di-*O*-*β*-glucoside (Figure 5) is the major anthocyanin in tepals, with a content of 9.68 mg g^−1^ dry weight according to Serrano-Díaz et al. [11].

Regarding the analytical techniques for *C.s.*, the flavonoid fraction in this spice was analyzed by liquid chromatography with photodiode array detection and mass detector in series (LC–DAD–MS/MS) [58]. In juices obtained from cold-pressed *C.s.*, floral bioresidues, kaempferols derivatives, and anthocyanins were determined by liquid chromatography with photodiode array detection (LC–DAD), using high-resolution mass spectrometry (LC–ESI–(HR)MS^n^) [73]. Goupy et al. [72] identified and quantified flavonols and anthocyanins in tepals by ultra-performance liquid chromatography coupled to diode array detection (UPLC–DAD) and ion trap mass spectrometry with either electrospray ionization (ESI–MS^n^). Further, an HPLC–DAD method was validated for the analysis of floral bioresidues form saffron spice production by Serrano-Díaz et al. [11]. Kaempferols of *C.s.* tepals were purified by flash column chromatography and identified by thin layer chromatography (TLC), HPLC–DAD, infrared (IR), and nuclear magnetic resonance (^1^H and ^13^C NMR) [71]. Further, due to the increasing importance of the metabolites of the floral bioresidues and the whole flower of *C.s.* as new products of application in the agri-food, nutraceutical, and cosmetics industry, the stability of the polyphenol content under different storage conditions was studied [75]. Moreover, the fact that, rather than a waste product of the saffron spice production, the tepals can be a readily exploitable good source of flavonols and anthocyanins for many applications (ranging from nutraceutical food supplements to cosmetic antiaging creams) is generating the continuous development of methods for determination of the compounds of *C.s.* flower.

Thereby, numerous analytical techniques are used to assess both bioactive compounds of saffron, floral bioresidues and even the whole flower of *C.s.* Regarding saffron quality, ISO 3632:2011 is the most relevant standard, which should be revised, and other more accurate analytical techniques that use validated methods could be included to guarantee an optimum evaluation of the compounds of interest in a detailed way. The growing interest of the bioactive molecules of floral bioresidues and the whole flower of *C.s.* means that today, they are the focus of new research and new analytical methods which are being developed.

## 3. Bioactivity of *Crocus sativus* L. Compounds

Saffron, in addition to being used in foods as flavoring and coloring, has been used as a natural preservative and for its possible pharmacological properties [6,36,76,77]. There are numerous scientific studies in the last 10 years that highlight the biomedical and pharmacological properties of saffron or some of its metabolites in the prevention and development of various chronic diseases [8,76,77,78,79,80].

Several researches explain the bioactivity of compounds present in saffron. However, it is known that in floral bioresidues, there are also bioactive metabolites [81].

Saffron is a source of carotenoids and natural pigments with a characteristic chemical structure of the system of conjugated double bonds and is responsible for the processes of color, reactivity, and energy transfer [82]. Due to its high richness in antioxidant compounds, it can be considered a nutraceutical. Antioxidant compounds with different chemical structures are present in both the stigma and the rest of the flower. Its action is developed by eliminating the generation of free radicals or indirectly increasing the endogenous cellular antioxidant defenses, for example, by activating the transcription factor pathway of the nuclear factor 2 derived from the erythroid (Nrf2). The generation of free radicals and the oxidative stress that produces cellular alterations are often cited as an important factor in the etiology of neurodegenerative diseases [83,84,85]. Alternative mechanisms of action have also been suggested for the neuroprotective effects of these compounds, such as the modulation of signal transduction cascades or effects on gene expression [83,84].

The phenolic compounds and the carotenoids are the main sources of antioxidant compounds in the diet. They protect us from the damage caused by reactive oxygen species (ROS) [86]. Saffron and its metabolites, crocins and safranal, have been shown to be powerful eliminators of ROS. Thus, some of the healthy effect of the spice can be explained [87,88,89,90,91].

The effects of the bioactive components are related to the bioavailable dose, not the dose ingested. Likewise, a bioavailable dose can cause different magnitudes of effects [92].

### 3.1. Bioactivity and Bioavailability of the Three Main Compounds of Saffron

The research attributes a potential therapeutic value to the three main components of saffron: crocins, picrocrocin, and safranal, present in the stigma [93]. Apart from its three main types of compounds, there are other carotenoids, carbohydrates, fiber, proteins, fats, vitamins (riboflavin and thiamine), minerals, and phenolic compounds such as anthocyanins and kaempferol glycosides, in addition to many other elements that confer nutritional and beneficial properties for health [5,93].

The pharmacokinetics of the most widespread carotenoids in nature are known. These are absorbed in the intestinal mucosa by passive diffusion, incorporated in the chylomicrons without being modified, and subsequently secreted into the bloodstream [94,95]. The main carotenoids of saffron have the peculiarity of their solubility in water due to the fact that they are glycosidic esters. Their absorption and metabolism are still to be solved [96]. Crocins and crocetin have low stability, poor absorption, and low bioavailability [97]. Crocins are not absorbed after oral administration, but they are hydrolyzed in an important way to crocetin in the intestinal tract [98]. From an aqueous extract of saffron, crocins and picrocrocin were bioaccessible (50% and 70%, respectively) under in vitro gastrointestinal digestion conditions [99]. A later study indicated that in spite of this high bioavailability, the quantities transported using a cellular transport model coupled with an in vitro digestion, 0.5% for crocins (2.93 × 10^−3^ g) and 0.2% for picrocrocin (1.44 × 10^−3^ g), were 10 times lower than those of the crocetin [100].

In vitro studies have shown that crocins of saffron are probably not bioavailable in the systemic compartment after oral application because they are rapidly hydrolyzed, mainly by enzymes of the intestinal epithelium and, to a lesser extent, by the gut microbiota, to deglycosylated *trans*-crocetin, which is absorbed by passive diffusion through the intestinal barrier [101]. It is interesting to observe, given that the pharmacological effect is attributed to crocetin, that the oral administration of 60 mg/kg of crocins, once daily for 4 days, reaches a concentration 56–81 times greater than crocetin in rat serum than the oral administration of crocetin. Thus, the oral administration of crocins is more beneficial than crocetin [84].

Linardaki et al. [102] demonstrated that after the administration of saffron extracts to mice, crocetin reaches the brain and, therefore, crosses the blood–brain barrier (BBB). *Trans*-crocetin is the only active metabolite that has been shown to cross the BBB and reach the central nervous system when pure crocetin or a saffron extract is administered. Yoshino et al. [103] showed that crocetin arrived at the maximum concentrations in plasma and brain 90 minutes after the oral administration of pure crocetin to rats and was significantly higher in the group that received crocetin as compared to the control group that did not receive crocetin. The pharmacokinetics of *trans*-crocetin have been validated in animal models [104,105,106] and in plasma from healthy volunteers [103]. Asai et al. [104] proposed that after oral administration of crocins, they are hydrolyzed to *trans* and *cis*-crocetin and thus incorporated into the blood circulation. Subsequently, *trans*-crocetin can be partially conjugated with mono and diglucuronides in the intestinal lumen, in the intestinal mucosa, and in the liver; and experiences an enterohepatic circulation. It was also shown that the pharmacokinetics of crocetin were proportional to the dose [107] and were studied in a compartmental model, comparing oral and intravenous administration of saffron extract (60 mg/kg) in mice. Crocetin levels in blood were higher after oral administration than in intravenous administration, probably due to the wide hydrolysis of the crocins in the intestinal lumen to *trans*-crocetin, which is rapidly absorbed by passing blood through the portal vein. The analysis of the tissues reveals an extensive distribution of crocetin in the liver and kidneys [108]. The advances in the knowledge of the bioaccesibility of bioactive compounds of saffron will contribute to developing their biomedical applications.

There are many scientific studies published in the last 10 years that highlight the biomedical and pharmacological properties of saffron or its metabolites [8,76,78,79]. These function on many systems of the body, such as the gastrointestinal, cardiovascular, endocrine, nervous, and immune system, which, among others, are attributed mainly to the carotenoids present in the spice, crocetin and crocins. Some authors attribute the antioxidant action indistinctly to any of the components of saffron [87,90,91], and others ascribed these effects to the total aqueous extract of saffron and crocins [109]. The bioactivity attributed to safranal presents great variability and is less known than that of crocins [100]. Safranal is especially attributed to be an antidepressant [110] and to have an anticonvulsant effect compared to crocins that did not show it [110,111]. Safranal combated oxidative stress in neurons, due to its antioxidant action, by eliminating free radicals [88]. Picrocrocin exhibited antiproliferative activity in the human cancer cell line [100].

It is attributed to saffron through the following actions: anti-inflammatory [112], anticonvulsant [111], satiating [113], antihistamine, anti-asthmatic, genoprotective, antitussive, and protective of the gastric mucosa and sexual dysfunction [8]. It is useful in promoting and regulating menstrual periods, and it soothes lumbar pains, which accompany menstruation [8,76,114,115].

Saffron and its metabolites have pharmacological effects on the central nervous system, with action on memory and learning, thus having effects on Alzheimer’s disease, Parkinson’s disease, cerebral ischemia, age macular degeneration, multiple sclerosis, and complications of diabetes mellitus; antidepressant and anxiolytic effects have also been reported [84,93,109,116,117,118,119,120].

The neuroprotective activity of saffron has been demonstrated in experimental models of cerebral ischemia [110,120,121], observing a reduction of oxidative damage in cerebral microvessels. Several studies claim that crocetin increases the diffusion of oxygen and, therefore, the oxygenation of various tissues by increasing oxygen in blood plasma [122,123,124,125,126]. In 2011, Yang et al. [127] demonstrated that crocetin, administered to experimental animals after hemorrhagic shock, significantly increased survival and reduced apoptosis. Therefore, crocetin could increase blood flow, which could have a positive effect on diabetes mellitus, because one of the most severe complications of this disease is attributed to neuronal damage caused by decreased blood flow. Moreover, saffron has shown neuroprotective actions in age-related macular degeneration; thus, in clinical trials at doses of 20 mg/day, significant protective effects against damage to the retina have been reported [128,129,130].

A randomized double-blind study in children and adolescents with attention deficit hyperactivity disorder (ADHD) compared the safety and efficacy of saffron versus treatment with methylphenidate to improve the symptoms. Twenty to thirty milligrams a day of saffron, depending on the weight of the child, were administered for 6 weeks, and there were no significant differences between the two treatments [131]. Thus, short-term therapy with saffron capsules showed the same efficacy compared with methylphenidate.

Saffron may have antioxidant and anti-amyloidogenic activity with a positive effect on cognitive function. Inhibitory effects of amyloid β-peptide fibrillogenesis or acetylcholinesterase activity, effective in Alzheimer’s disease, with efficacy compared to donepezil, have also been demonstrated at doses of 30 mg/day [93,132,133,134].

Crocetin may be useful in the prevention of Parkinson’s disease [83], protecting many of the cells of the substantia nigra pars compacta.

Saffron and its metabolites have been shown to be effective in different models of psychiatric disorders, including depression and anxiety, comparing their effects with imipramine or fluoxetine [135,136,137,138,139]. A study showed that there are no significant differences between the use of saffron and the drug [140]. This action is attributed to the crocins that can act through the inhibition of the absorption of the neurotransmitters dopamine and noradrenaline, and safranal, inhibiting the reuptake of serotonin. There are studies in vivo that suggest inhibitory effects on monoamine-oxidases (MAO-A and MAO-B), which are degradation enzymes of the aforementioned neurotransmitters, causing an increase in their levels in the synaptic space and decreasing depressive symptoms of patients [141].

Antineoplastic effects have been observed in models of cancer cells and in rats. The aqueous extract of saffron inhibits the progression of different types of cancer, such as gastric [142], colorectal [143], pancreatic [144], and bladder [145], by reducing cell growth and inducing apoptosis of cancer cells [146]. Recent studies carried out in in vitro and in vivo models indicate that the components of saffron, particularly crocins and crocetin, can have an anticarcinogenic effect in breast, lung, and pancreas cancer cells [147]. The mechanism of action to date is unclear, but its activity could be related to the antioxidant action of the crocins, which could affect the regulation of cell growth and modulate gene expression and the immune response [148]. Different studies indicate its action as an activator of cellular apoptosis in cancer cells, not affecting the normal ones [142,149,150,151]. Picrocrocin reduced the proliferation of adenocarcinoma and hepatocarcinoma cells in humans [100].

In animal and cell culture models, crocetin is responsible for the inhibition of nucleic acid synthesis, improving the activity of the endogenous antioxidant system, inducing apoptosis, and damaging the signaling pathways of tumor growth factor [152].

Several studies show the potential effect of saffron in the treatment of diseases of the cardiovascular system. As mentioned above, many studies report that crocetin increases the diffusion of oxygen; therefore, the oxygenation in various tissues can increase, since the diffusion oxygen in the blood plasma increases. Thus, in atherosclerosis, crocetin increases the diffusion of oxygen in blood plasma, offsetting the decrease caused by a high level of cholesterol, which has been shown to have an effect against arteriosclerosis and act as a reducer of blood cholesterol levels [153,154,155]. The increase in oxygenation of various tissues, due to crocetin and *trans*-sodium crocetinate, has also been studied [122,123,124,125,126,156,157].

There are studies that claim that it is possible to lose weight by consuming saffron [113], suggesting several mechanisms: blocking the digestion of fats in the diet through the inhibition of pancreatic lipase, the suppression of inflammatory cytokines, and adipocyte differentiation; decrease in food intake by increasing satiety, or the feeling of fullness due to the elevation of the level of neurotransmitters or hormonal functions; and the increase in glucose and the metabolism of lipids.

Hepatoprotective effects are described in in vivo studies, where crocins attenuate the activation of caspases, essential mediators of apoptosis or cell death, and the reduction of the ratio between the proapoptotic protein bax and the antiapoptotic Bcl-2 (bax / Bcl-2), which reduces hepatotoxicity [158].

In conclusion, numerous studies about biomedical and pharmacological properties of saffron and its bioactive molecules have been conducted in the last 10 years. Table 2 shows the main bioactivity of saffron and its compounds of interest. The potential effects of saffron and its bioactive compounds depend on the amount consumed and its bioavailability. Thus, further research to clarify this relevant aspect is necessary, since this knowledge is the interest of industry, and this will contribute to developing biomedical applications. In addition, it is worth noting that the oral administration of crocins has the advantage over crocetin, and crocetin may be the active component potentially responsible for the pharmacological effect of crocins.

### 3.2. Bioactivity and Bioavailability of Phenolic Compounds of Crocus sativus L. Flower

The main byproduct of saffron production, the flower tepals of *C.s.*, contains minerals; alkaloids; anthocyanins; kaempferol, and their glycosides, mainly. This composition confers antioxidant properties and its possible pharmacological application [81,159,160]. High phenolic content was found in whole flowers, in floral bioresidues, and in tepals [1,11,72,75]. Five derivatives of kaempferol in this spice have been identified [58]. The highest content of phenolic compounds is mainly in the tepals [14]. Serrano-Díaz et al. [161] demonstrated the absence of cytotoxicity in an aqueous extract; therefore, this extract can be added in foods.

In traditional medicine, the tepals of *C.s.* flower were consumed as an antispasmodic, stomach, anxiolytic, antitumor, and antidepressant [81]. There are different studies that indicate the antioxidant activity due to the phenolic content of the tepals [72,74,159,162,163]. The stamens and perianth have shown important antifungal, cytotoxic, and antioxidant activities [164]. In the pollen, kaempferol, kaempferide, and isohamnetin have been detected, which are methylated glycosides with antityrosinase activity [9].

Tepal extracts of *C.s.* flower have been reported to have hepatoprotective effects, since ameliorates induced acute liver injury in rats, returning blood parameters and the histopathology of liver to almost normal levels [165], and they also showed ameliorative effects on kidney failures induced in rats [166]. Another study about the effects of tepals extracts of *C.s.* flower on blood parameters, immune system, and spleen histology showed an increase in antibody response without any change in hematological parameters and spleen histology [167]. Thus, tepal extracts could be used for biomedical proposes.

Moreover, tepal extracts, as a promising source of antioxidants, have been used as ingredients in high-end cosmetic products to develop new dermal treatments, since they could play a role in the process of aging [13].

Antioxidant, anti-inflammatory, neuroprotective, and anticancer actions are attributed to kaempferol [68,168,169,170,171]. It should be noted that in a recent study, the kaempferol aglycone showed potent antitumor effect compared to the glycosides kaempferol 3-*O*-*β*-glucoside and kaempferol 3-*O*-*β*-rutinoside. The absence of the glucosyl groups could be the explanation of this fact [170]. Kaempferol can inhibit the inflammatory response induced due to endotoxins in acute lung injury in mice as well as in cellular models [172]. The inhibition of the expression of inflammatory mediators was demonstrated in a cellular model of intestinal inflammation in rats [173]; further, anti-inflammatory effects were observed on the inflammation induced by *Helicobacter pylori* [174]. In addition, antidepressant effects [175] and improvement of wound healing in diabetic and non-diabetic rats [176] have also been attributed to kaempferol.

The kaempferol 3-*O*-β-sophoroside, one of the main components found in floral bioresidues, has anti-inflammatory activity, which has been demonstrated in human endothelial cells, which could be useful as a therapy for vascular inflammatory diseases [177]. Analgesic action has also been attributed to it in tests with mice [178].

Apart from kaempferol, quercetin 3-*O*-*β*-sophoroside is another flavonol present in the tepals of *C.s.* flower [11]. Quercetin is mainly a classic candidate for anticancer drug design, due to the opposing effects that it exerts on different signaling networks to inhibit cancer progression [179]. A study of the efficacy of quercetin on oral squamous cell carcinoma (OSCC) in cultured OSCC cells suggested that this metabolite could have potential as a new chemopreventive agent or serve as a therapeutic adjuvant for OSCC [180]. Another study suggests that quercetin could prevent prostate cancer, since it acts as a chemopreventive agent in preclinical models of prostate cancer [181]. Thus, this flavonol could be a potential biocompound for disease prevention and therapy [182].

The glucosides of the delphinidin, together with other anthocyanins, can act by preventing the progression of the tumor, due to its ability to inhibit angiogenesis [183].

The principal bioactivity of the phenolic compounds of saffron and floral bioresidues of *C.s.* flower, mentioned above, are shown in the following table (Table 3).

The beneficial effects of phenolic compounds will depend on their absorption and distribution to tissues and cells. There are considerable differences in the pharmacokinetics of the different types of flavonoids. The absorption ranges in the small intestine are from 0% to 60% of the average dose, and their elimination half-life ranges from 2 to 28 hours [184].

The bioavailability of phenolic compounds is influenced by their structure. Phenolic compounds exist as free aglycones and in the form of glycosides. The aglycones and polyphenols bound to glucose, galactose or xylose are absorbed in the small intestine after enzymatic deglycosylation by a diffusion mechanism. The main sites of metabolism of the flavonoids are the liver and the flora of the colon. In the liver, *O*-methylation, sulfation, and glucuronidation of hydroxyl groups are produced, improving flavonoid absorption [185].

Most in vivo studies show good gastric absorption of aglycones such as quercetin and daidzein, while glycosides are poorly absorbed. The aglycone isoflavones are absorbed in the stomach, while their glycosides are absorbed in the intestine. Anthocyanins are an exception: Their glycosides appear intact in blood, which suggests the existence of a specific mechanism of anthocyanin absorption at the gastric level, which could involve transport through gastric bilitranslocase [185].

There are few studies in which the bioavailability of different flavonols have been compared, and their results suggest that kaempferol is absorbed more efficiently than quercetin, and quercetin is more extensively metabolized to other compounds [186,187,188]. Kaempferol was found to be stable at a wide range of different pH values, while quercetin required an acid pH to prevent its oxidative degradation [188].

The pharmacokinetics of kaempferol were studied in rats after oral and intravenous administration, confirming an absolute bioavailability of kaempferol of 11.0% [189]. Kaempferol has low solubility in water and, therefore, low oral bioavailability [169].

Thereby, the study of the bioactivity of the phenolic compounds of saffron; of tepal extracts of *C.s*. flower, of its flavonols in a detailed manner, mainly kaempferol and quercetin; as well as the anthocyanins has gained interest in recent years, due to their potential antioxidant activity and pharmacological properties. However, further research is necessary to understand the health effects of these phenolic compounds present in floral bioresidues and whole flower of *C.s*., as well as in saffron. Moreover, due to its relevance, it is necessary to carry out more studies to clarify its bioavailability.

## 4. Conclusions and Future Perspectives

Most of the *C.s.* literature has been based on the study of its stigmas, because they are highly valued as a spice. Today, the floral bioresidues of *C.s.* represent an interesting source of phenolic compounds, which together with the bioactive compounds present in the stigma make this flower a potent supply of antioxidant compounds. Further, the possibilities of mechanization and modernization of *C.s.* offer a greater interest for this crop, again increasing its cultivation.

Several analytical techniques are used to assess the saffron quality. The most relevant one is the ISO 3632:2011 standard, which is employed in international commercial agreements, but there are numerous studies which suggest that such a standard should be revised, especially for analyzing safranal, and others analytical techniques which are more accurate, such as HPLC–DAD or NMR, could be included. On the other hand, at present, there is an important trend regarding the determination of the bioactive compounds of floral bioresidues and whole flower of *C.s.* In this case, the analytical techniques employed are adequate and allow assessing the content of these bioactive molecules. Due to this increasing interest, new methods are being developed. Moreover, the stability of these bioactive compounds has been studied, but other different storage conditions of interest for the industry could be also researched.

Saffron’s properties have been known since antiquity, and the bioactivity of its metabolites has been studied along time. Thus, in recent years, the study of its bioavailability is the purpose of many studies, but these give different and sometimes contradictory results, so it is necessary to continue this research line, since its knowledge will contribute to developing biomedical applications.

The bioactivity of the phenolic compounds of floral bioresidues of *C.s.* has been studied to a lesser extent than saffron. Flavonols, mainly kaempferol, quercetin, and their glycosides, are gaining interest due to their antioxidant activity, which reports beneficial pharmacological properties. It should be noted that high quantities of kaempferols from the tepals of *C.s.* flower can be extracted, which are powerful anti-inflammatory agents. However, further research on their health effects and bioavailability is necessary.

The high variety of different flavonoids, including anthocyanins, kaempferol, quercetin, and their glycosides, along with the large amounts of crocins, picrocrocin, safranal, and crocetin, confirms the interest of the use of floral bioresidues and whole flower of *C.s.* as potential sources of natural antioxidants, which can be considered as active ingredients in food supplements, functional foods, and beverages; and in pharmaceutical preparations, cosmetic formulations, and as ingredients *Crocus sativus* L.-based of the herbal medicine.

## Figures and Tables

**Table 1:** Analytical techniques for saffron analysis.

Analytical Technique [Reference]	Indicative Data or Analyte	Information
Spectrophotometry UV-vis [15]	Coloring strength, $E_{1\;cm}^{1\%}$257 nm and $E_{1\;cm}^{1\%}$330 nm	ISO 3632:2011
HPLC–DAD [46,47]	Crocins, picrocrocin, and safranal	Saffron quality
LC/DAD/MS/MS [58]	Crocins, picrocrocin, and flavonoids	Identification of saffron metabolites
UHPLC–MS/MS [59]	Crocins	Differentiation of the obtaining process of saffron
NMR [55]	Saffron compounds	Metabolic fingerprinting
DHS–GC–MS [38]	Safranal and other volatile compounds	Quality of aroma
e-Nose [60]	Volatiles of saffron as a whole	Determination geographical origin
PTR–TOFMS [61]	Volatile compounds	Quality of aroma
Raman spectroscopy [62]	Sum of crocins and coloring strength	Saffron quality
NIR spectroscopy [63]	Control of saffron quality	Saffron Quality/Geographical origin
Derivatization–HPLC–DAD [64]	Free amino acids and ammonium	Determination of geographical origin
MIR spectroscopy [65]	FT-IR spectra saffron filaments	Determination of geographical origin
Tristimulus colorimetry [66]	Color	Saffron quality

**Table 2:** Summary of the main bioactivity of saffron and its bioactive molecules.

Bioactive Compound	Bioactivity [Reference]	Model	Dose
*trans*-crocetin	Cross the blood–brain barrier and reach the central nervous system [102,103]	Rats	Oral administration (100 mg/kg)
Crocetin	Neuroprotection [83]	Hemi-Parkinson rats	Peripheral administration (25, 50 and 75 µg/kg body weight)
	Improved post-shock survival and reduced apoptosis [127]	Rats	Bolus injection (2 mg/kg body weight)
	Cardioprotective effects (after myocardial ischemia reperfusion injury) [121]	Adult male Wistar rats	Intragastric administration (50 mg/kg/day)
Crocins	Hepatoprotective effects [158]	Rats	Intraperitoneally (25 mg/ kg body weight/day for 4 weeks)
Safranal	Antidepressant [110]	Rats	Peripheral administration (15.5 mg/kg body weight.)
	Anticonvulsant [111]	Mice	Injected (0.15 and 0.35 mg/kg)
Picrocrocin	Antitumor effects [100]	Human colon adenocarcinoma (Caco-2-cell model)	8–24 µM
Saffron extracts	Satiating [113]	Human (randomized, double-blind, placebo-controlled, parallel-group)	Oral administration (capsule: 176.5 mg extract/day for 8 weeks)
	Reduce cognitive deterioration (Alzheimer’s disease) [134]	Patients (randomized double-blind parallel-group)	Oral administration (capsule: 30 mg/day for 12 months)
Saffron	Premenstrual syndrome [115]	Women (double-blind, randomized and placebo-controlled trial)	Oral administration (capsule: 30 mg/day for 6 months)
	Neuroprotection (macular degeneration) [130]	Albino rats with light-induced photoreceptors degenerations	Oral administration (1 mg/kg/day for 6 weeks)
	Improve the symptoms of children with deficit hyperactivity disorder [131]	Children	Oral administration (20–30 mg/ day for 6 weeks)
	Effective treatment in depression and anxiety [140]	Patients (double-blind controlled clinical trial)	Oral administration (30 mg/day per 6 weeks)

**Table 3:** Summary of the main bioactivity of phenolic compounds of saffron and floral bioresidues of *Crocus sativus* L. flower.

Bioactive Compound	Bioactivity [Reference]	Model	Dose
Tepal (ethanol; 80%) extracts of *C.s.* flower	Hepatoprotective effects [165]	Rats	Administration by oral gavage (20 mg/kg body weight for 6 days)
	Ameliorative effects on kidney failures [166]	Rats	Intraperitoneal injection (40 mg/kg body weight for 7/13 days)
	Increase antibody response [167]	Rats	Intraperitoneal injection (75 mg/kg body weight for 14 days)
Kaempferol aglycone	Antitumor effects [170]	Colon cancer cells	75 µM
	Anti-inflammatory effects in acute lung injury [171]	Cell	100 µM
		Mice	Intraperitoneal injection (50 mg/kg body weight)
	Anti-inflammatory effects [173]	Cellular model of intestinal inflammation in rats	12.5, 25 and 50 µM
	Anti-inflammatory effects on *Helicobacter pylory*-induced inflammation [174]	Gastric adenocarcinoma cell	6.25, 12.5, and 25 µM
	Antidepressant effects [175]	Chronic social defeat stress mouse model	Intraperitoneal injection (20 mg/kg body weight)
	Wound healing effects [176]	Incisional and excisional wound models on diabetic and nondiabetic rats	Topically applied (1% weight/weight for 14 days)
Kaempferol 3-*O*-*β*-sophoroside	Anti-inflammatory effects [177]	Human endothelial cells	>0.05 µM
	Analgesic effects [178]	Mice	Intraperitoneal injection (50 mg/kg body weight)
Quercetin	Chemopreventive effects. Inhibit cell growth and invasion/migration of the cells [180]	Cultured oral squamous cell carcinoma cells	2 mg/mL
	Chemopreventive effects [181]	Male Sprague Dawley rats	Oral administration (200 mg/kg body weigh/ trice a week for 16 weeks)
Delphinidin 3-*O*-*β-*glucoside	Prevent tumor progress by inhibiting angiogenesis and cell migration [183]	Breast cancer cells	200 µM
